# Diabetic Lipemia

**Published:** 1911-10

**Authors:** 


### Diabetic Lipemia

Klemperer, *Med. Woch.,* December 23, 1910, calls attention to lactescent blood serum as an indication of excess of fats and, still more, of lipoids such as lecithin and cholesterin. In ninety-two diabetics without acidosis, it was absent, whereas it was present in thirty-nine of fifty cases with acidosis.

